# Dynamics of clonal hematopoiesis under DNA-damaging treatment in patients with ovarian cancer

**DOI:** 10.1038/s41375-024-02253-3

**Published:** 2024-04-18

**Authors:** Christopher Maximilian Arends, Klara Kopp, Raphael Hablesreiter, Natalia Estrada, Friederike Christen, Ute Martha Moll, Robert Zeillinger, Wolfgang Daniel Schmitt, Jalid Sehouli, Hagen Kulbe, Maximilian Fleischmann, Isabelle Ray-Coquard, Alain Zeimet, Francesco Raspagliesi, Claudio Zamagni, Ignace Vergote, Domenica Lorusso, Nicole Concin, Lars Bullinger, Elena Ioana Braicu, Frederik Damm

**Affiliations:** 1 Department of Hematology, Oncology, and Cancer Immunology, Charité–Universitätsmedizin Berlin, corporate member of Freie Universität Berlin and Humboldt-Universität zu Berlin, Berlin, Germany; 2 Department of Pathology, Stony Brook University Cancer Center, Stony Brook, NY 11794 USA; 3 Department of Obstetrics and Gynaecology, Molecular Oncology Group, Comprehensive Cancer Center-Gynaecologic Cancer Unit, Medical University of Vienna, Vienna, Austria; 4 Department of Pathology, Charité - Universitätsmedizin Berlin, corporate member of Freie Universität Berlin and Humboldt Universität zu Berlin, Berlin, Germany; 5 Department of Gynaecology, European Competence Center for Ovarian Cancer, Charité-Universitätsmedizin Berlin, Corporate Member of Freie Universität Berlin, Humboldt-Universität zu Berlin, and Berlin Institute of Health, Campus Virchow Klinikum, Berlin, Germany; 6 North Eastern German Society for Gynecological Cancer. Tumor Bank Ovarian Cancer Network, Berlin, Germany; 7 Klinik für Innere Medizin II, Abteilung Hämatologie und Onkologie, Universitätsklinikum Jena, Jena, Germany; 8 Centre Anticancereux Léon Bérard, University Claude Bernard Lyon, GINECO Group, Lyon, France; 9 Department of Obstetrics and Gynecology, Medical University of Innsbruck, Austrian AGO, Innsbruck, Austria; 10 Gynecologic Oncology Unit Fondazione IRCCS Istituto Nazionale Tumori, Milan, Italy; 11 Division of Oncology, IRCCS Azienda Ospedaliero-Universitaria di Bologna, Bologna, Italy; 12 Division of Gynecological Oncology, Department of Gynecology and Obstetrics, Leuven Cancer Institute, Katholieke Universiteit Leuven, Leuven, Belgium; 13 Belgium and Luxembourg Gynaecological Oncology Group (BGOG), Leuven, Belgium; 14 Humanitas San Pio X, Humanitas University Rozzano, Milan, Italy; 15 German Cancer Consortium (DKTK) and German Cancer Research Center (DKFZ), Heidelberg, Germany

**Keywords:** Stem-cell research, Cancer genomics, Cancer genetics

## Abstract

Clonal hematopoiesis (CH) driven by mutations in the DNA damage response (DDR) pathway is frequent in patients with cancer and is associated with a higher risk of therapy-related myeloid neoplasms (t-MNs). Here, we analyzed 423 serial whole blood and plasma samples from 103 patients with relapsed high-grade ovarian cancer receiving carboplatin, poly(ADP-ribose) polymerase inhibitor (PARPi) and heat shock protein 90 inhibitor (HSP90i) treatment within the phase II EUDARIO trial using error-corrected sequencing of 72 genes. DDR-driven CH was detected in 35% of patients and was associated with longer duration of prior PARPi treatment. *TP53*- and *PPM1D*-mutated clones exhibited substantially higher clonal expansion rates than *DNMT3A-* or *TET2-*mutated clones during treatment. Expansion of DDR clones correlated with HSP90i exposure across the three study arms and was partially abrogated by the presence of germline mutations related to homologous recombination deficiency. Single-cell DNA sequencing of selected samples revealed clonal exclusivity of DDR mutations, and identified DDR-mutated clones as the origin of t-MN in two investigated cases. Together, these results provide unique insights into the architecture and the preferential selection of DDR-mutated hematopoietic clones under intense DNA-damaging treatment. Specifically, PARPi and HSP90i therapies pose an independent risk for the expansion of DDR-CH in a dose-dependent manner.

## Introduction

Clonal hematopoiesis (CH), characterized by the expansion of somatically mutated hematopoietic stem cells, has emerged as a prevalent phenomenon associated with the development of hematologic malignancies [1–4], increased cardiovascular risk [5, 6] and other age-related pro-inflammatory conditions [7–11]. While in the unselected aging population somatic mutations in epigenetic regulator genes such as *DNMT3A*, *TET2* and *ASXL1* (collectively called DTA mutations) predominate, exposure to cytotoxic treatment, in particular platinum-based treatment, differentially selects for gene mutations affecting the DNA damage response (DDR) machinery, mainly *TP53*, *PPM1D*, *ATM* and *CHEK2* [12]. These clones show increased resistance to DNA damage-induced apoptosis, providing them with a selective advantage under the evolutionary pressure of cytotoxic stress [13–16]. Increasing evidence suggests that cancer patients with pre-existing mutant *TP53* clones are at elevated risk for developing therapy-related myeloid neoplasms (t-MNs) [12, 17–23].

In recent years, poly(ADP-ribose) polymerase (PARP) inhibitors (PARPi) have revolutionized the therapeutic landscape for patients with ovarian cancer, offering improved treatment outcomes and prolonged progression-free survival [24–29]. PARPi selectively target PARP enzymes and prevent the repair of DNA single-strand breaks. In cells with homologous recombination (HR) deficiency (HRD), for instance due to disruptive mutations in *BRCA1/2*, the accumulation of unresolved DNA damage leads to the formation of double-strand breaks during DNA replication and consecutive cell death [30, 31]. Several studies and meta-analyses have indicated that, similar to platinum treatment, PARPi treatment is associated with the occurrence of t-MNs [32, 33]. While emerging evidence suggests a role for CH in the development of t-MN under PARPi treatment [34, 35], a comprehensive understanding of the interplay between the dynamics of CH clones and PARPi therapy is lacking.

In this study, we performed an in-depth DNA sequencing analysis of CH in whole blood (WB) and plasma of patients with relapsed HGOC participating in the EUDARIO trial to elucidate the mutational landscape of CH, its associations with clinical outcomes, and the clonal architecture and evolutionary dynamics of CH clones under treatment. The prospective and randomized design of the EUDARIO trial allowed us to systematically examine clonal dynamics under intense DNA-damaging treatments including carboplatin, PARPi, and heat-shock protein 90 (HSP90) inhibition (HSP90i). Specifically, we show that PARPi and HSP90i therapy independently pose an increased risk for the development of DDR-CH in a dose-dependent manner in the setting of carboplatin.

## Methods

### Patients and materials

The European Trial on Enhanced DNA Repair Inhibition in Ovarian Cancer (EUDARIO)/European Network of Gynaecological Oncological Trials groups (ENGOT)-ov48 (NCT03783949; in the following called EUDARIO) is a multicenter, randomized, open-label, phase ll trial performed in women with relapsed, platinum-sensitive HGOC according to the ENGOT Model A [36]. By definition, patients had experienced progressive disease > 76 months after previous platinum-based treatment. A total of 120 women of age older than 18 years were randomized 1:1:1 to 3 different treatment arms. The backbone of all treatment arms consisted of 6 cycles of carboplatin-based chemotherapy followed by maintenance therapy with Niraparib. In the 2 experimental arms, the study drug Ganetespib, a HSP90 inhibitor [37], was administered during chemotherapy (arm B) or during chemotherapy and maintenance (arm C; see Supplementary Table S1 for a detailed treatment plan). The primary endpoint of the study was progression-free survival (PFS), secondary outcome measures included overall survival (OS), adverse events and best response. Peripheral WB and plasma specimens of EUDARIO participants were collected at the initiation of study treatment, at the initiation of maintenance PARPi treatment, and at the end of study treatment. DNA was extracted using commercially available kits as detailed in the Supplementary Methods section [38]. For single-cell sequencing analysis, additional 37 patients with OC receiving PARPi and/or platinum treatment were prospectively recruited at the Department of Gynecology, Charité Berlin, Germany. Written consent was obtained from all patients in accordance with the Declaration of Helsinki and the study was approved by local ethics committees.

### Targeted sequencing

A custom targeted sequencing panel (TWIST Bioscience, South San Francisco, CA, USA) was designed including 24 genes recurrently mutated in CH, 21 genes recurrently mutated in myeloid malignancies, as well as 27 additional genes involved in the HR pathway (Supplementary Table S2). Library construction for next generation sequencing was performed using a commercially available hybrid-capture based library preparation kit (TWIST Bioscience), in combination with custom sequencing adapters including 9 bp unique molecular identifiers (UMIs, xGen UDI-UMI adapters by Integrated DNA Technologies, Iowa, USA) to enable bioinformatic error-correction. Libraries were sequenced on the NovaSeq 6000 platform (Illumina, San Diego, CA, USA) in paired-end mode.

The sequencing data was processed using our in-house snakemake [39] pipeline, following previously described methods [40–43]. Briefly, consensus reads containing at least 3 raw reads were aligned to the GRCh38 reference genome using the BWA-MEM algorithm. Variant calling was performed using VarDict with a minimum allele frequency of 0.1% [44] and annotated with public databases using ANNOVAR [45]. Nonsynonymous variants in the coding region and splice site variants with a minimum alternate allele count of 10 consensus reads were retained. Variants with VAF > 45% were classified as germline mutations. Furthermore, variants with VAF > 40% that were reported in the dbSNP database as single nucleotide polymorphisms (SNPs) or had a population-based allele frequency (AF) > 1% in the gnomAD database, were classified as SNPs and excluded. Highly recurrent single nucleotide variants (*DNMT3A* codon R882, *GNB1* K57E, *JAK2* V617F, *SF3B1* codons K666 and K700, *SFRS2* codon P95, and *U2AF1* codons S34 and Q157) were retained.

For the analysis of paired WB and cfDNA samples, variants that were detected in either source with a VAF ≥ 0.8%, or in both sources with sum of the VAFs ≥ 0.8% were included in further analyses. Variants in cfDNA were classified as of non-hematopoietic origin if the VAF in cfDNA was 5-fold higher than in WB DNA. In the serial sample analysis in WB DNA and cfDNA, variants that were detected in at least 2 timepoints and with VAF ≥ 0.8% in at least one timepoint were retained. For variants not detected in all available timepoints sequencing data was manually reviewed for the presence of the variant beneath the variant calling threshold (0.1%), and, if present, the VAF was manually set to the detection threshold of 0.1%.

### Germline homologous recombination deficiency

WB DNA variant calls in *BRCA1* and *BRCA2* were annotated with the BRCAExchange database [46]. All variants with a VAF > 40% that were classified as pathogenic or likely pathogenic by ClinVar [47], the evidence-based network for the interpretation of germline mutant alleles (ENIGMA) [48] or expert review, or that were deleterious (splice site, truncating indel, stopgain, or startloss mutation) and had a population based AF < 1% were categorized as pathogenic germline *BRCA* mutations. For the remaining HR-related genes (as defined in Supplementary Table S2), variants with a VAF > 40% with deleterious mutations and a population-based AF < 1% were classified as pathogenic germline HRD mutations.

### Clonal fitness analysis

Clonal fitness was determined from paired samples as previously described [41, 49]. We modeled clonal growth over time as a sigmoid function

$$v(t)=\frac{1}{2}\frac{1}{{1+Ae}^{-{st}}},$$

where *v* is the VAF as a function of time *t* (in years), *A* is a numeric constant such that *v*(t = 0) equals the VAF at the first timepoint and *s* is a parameter quantifying the clonal fitness. Clones with a fitness *s* < −0.25/year were categorized as decreasing, clones with a fitness *s* > 0.25/year as increasing, and all others as stable.

### Single-cell sequencing analysis

Single-cell DNA sequencing of WB mononuclear cell samples was performed on the MissionBio Tapestri platform using the Tapestri Single-Cell DNA Sequencing V2 kit (MissionBio, South San Francisco, CA, USA) and the MissionBio Myeloid or Myeloid Koichi Takahashi (MDACC) [50] panels, depending on best amplicon coverage of the previously identified patient’s somatic mutations. In some cases, a sample-multiplexing approach was applied in which the individual SNP profile was used as specific sample marker, enabling sample pooling and bioinformatic decoding (see Supplementary Methods). The libraries were sequenced on the NovaSeq 6000 platform (Illumina, San Diego, CA, USA) using the S1 flow-cell for a 150 bp paired-end run with a 15% ratio of PhiX DNA. Sequencing reads were processed using Mission Bio’s Tapestri pipeline v2.0.2 with default settings and a customized analysis pipeline as detailed in the Supplementary Methods section. Downstream analysis was conducted on samples with a minimum of 400 genotyped cells. Clones in each sample were reported if the mutated allele was present in at least 5 cells.

### Statistical analysis

The statistical analysis was performed in R version 4.2.3. PFS and OS were assessed with crude Kaplan-Meier analysis and with multivariate Cox regression models including treatment arm, age, prior PARPi treatment, number of prior treatment lines, and *BRCA* status as covariates. Due to the exploratory nature of this study no prior sample size calculation was performed. Pairwise comparisons of variables were performed using Wilcoxon rank-sum tests or Fisher’s exact tests. The 2-sided level of significance was set at a *p*-value of < 0.05 without adjustment for multiple testing, if not stated otherwise.

## Results

### Detection of clonal hematopoiesis in peripheral whole blood DNA

**Fig. 1:**
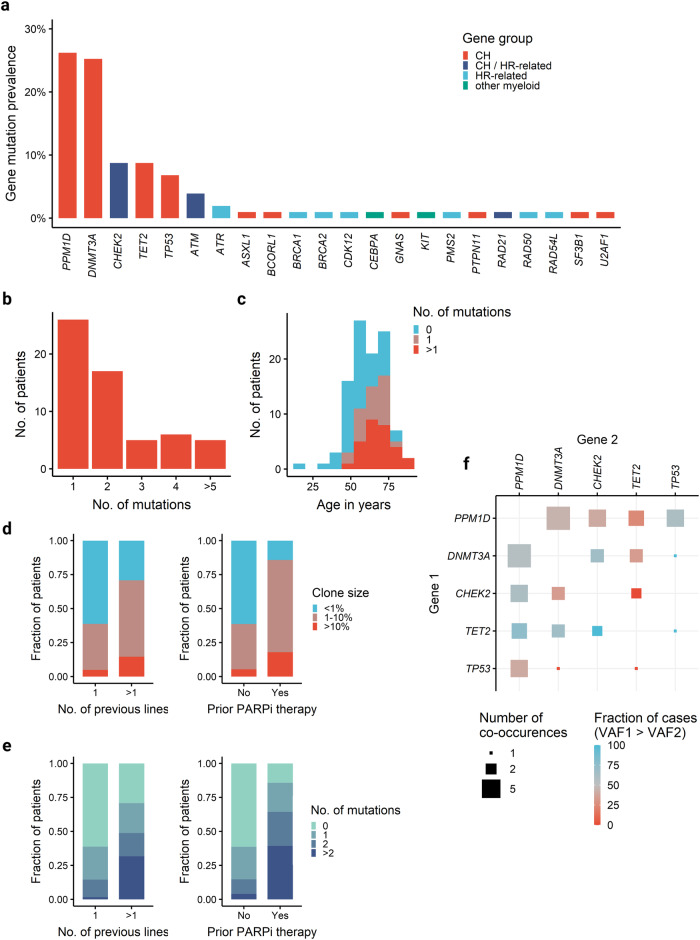
Detection of clonal hematopoiesis in whole blood DNA. Somatic mutation analysis on whole blood DNA from 103 patients enrolled in the EUDARIO study. Targeted sequencing of 72 genes listed in Supplementary Table S2. **a** Gene-specific prevalence of somatic mutations colored by gene class. **b** Number of patients with single and multiple mutations. **c** Age-related prevalence of CH mutations. **d** Stacked bar plot showing the clone size of the largest clone in relation to the number of prior treatment lines (left) and prior PARPi treatment (right). **e** Stacked bar plot showing the number of mutations per patient in relation to the number of prior treatment lines (left) and prior PARPi treatment (right). f) Analysis of mutation co-occurrence in patients with multiple mutations. The size of each square denotes the number of co-occurrences. Color depicts the fraction of cases in which gene 1 (y-axis) has a higher VAF than gene 2 (x-axis).

A total of 103 patients enrolled in the study had WB samples available at the initiation of treatment. WB targeted DNA sequencing identified 130 somatic mutations in 59 patients (57%) with a VAF of ≥ 1% (Supplementary Table S3a). Among these mutations, 24 affected HR-related genes, 8 of them in genes not commonly mutated in CH (Fig. 1a). In contrast to the mutational profile observed in healthy individuals where *DNMT3A*, *TET2*, and *ASXL1* (DTA) are most frequently mutated [51, 52], our analysis revealed a high prevalence of mutations in DDR genes, i.e. in *PPM1D* (51 mutations in 27 patients), *CHEK2* (11 mutations in 9 patients), *TP53* (7 mutations in 7 patients), and *ATM* (4 mutations in 4 patients). A total of 30 patients (29%) harbored multiple mutations (Fig. 1b), with 6 patients exhibiting 5 or more mutations. Notably, 9 patients had multiple mutations in *PPM1D*. Age (Fig. 1c), number of prior therapy lines (1 *vs* > 1 therapy line), and prior PARPi treatment were significantly associated with the presence of CH (Table 1). Specifically, the number of previous therapy lines and prior exposure to PARPi treatment were strongly associated with both clone size and number of mutations (Fig. 1d, e). In a multivariate logistic regression with age, number of previous therapy lines, and duration of prior PARPi treatment as independent variables, age (odds ratio [OR] 1.95, 95%-CI 1.21–3.12 per decade, *p* = 0.006) was the only significant predictor for the presence of DTA mutations (Supplementary Table S4a). In contrast, age (OR 1.98, 95%-CI 1.19–3.27 per age decade, *p* = 0.008), number of previous therapy lines (OR 3.18, 95%-CI 1.12–9.10, *p* = 0.030) and duration of prior PARPi therapy (OR 1.09, 95%-CI 1.01–1.17 per month, *p* = 0.032) were significant predictors for the presence of DDR mutations (Supplementary Table S4b). These data strongly suggest that mutations affect different genetic pathways in therapy-related CH *versus* ageing-related CH.

**Table 1:** Demographic and clinical baseline characteristics of 103 participants of the EUDARIO cohort.

Characteristic	Level	CH negative *n* = 50	CH positive *n* = 53	*p*-value
**Age in years – median (IQR)**		55 (5066)	68 (6073)	< 0.001
Treatment arm – No. (%)	A	16 (32.0)	18 (34.0)	1.00
	B	17 (34.0)	18 (34.0)	
	C	17 (34.0)	17 (32.1)	
ECOG performance status – No. (%)	0	44 (88.0)	39 (73.6)	0.08
	1	6 (12.0)	14 (26.4)	
*BRCA* status – No. (%)	Mutated	21 (42.0)	14 (26.4)	0.14
	Wildtype	27 (54.0)	33 (62.3)	
	Unknown	2 (4.0)	6 (11.3)	
History of cancer – No. (%)	No	48 (96.0)	45 (84.9)	0.09
	Yes	2 (4.0)	8 (15.1)	
Number of previous lines – No. (%)	1	38 (76.0)	24 (45.3)	0.002
	> 1	12 (24.0)	29 (54.7)	
Prior PARPi treatment – No. (%)	No	46 (92.0)	29 (54.7)	< 0.001
	Yes	4 (8.0)	24 (45.3)	
Platelet count – median (IQR)		246 (214298)	258 (180–368)	0.768
White blood cell count – median (IQR)		7.00 (5.62–8.34)	6.33 (5.07–8.50)	0.342
Hemoglobin – median (IQR)		12.95 (12.28–13.60)	12.30 (11.40–13.00)	0.004

CH was defined as the presence of a somatic mutation in a typical CH gene (as defined in Supplementary Table S2) with a VAF ≥ 1%. *ECOG* Eastern cooperative oncology group, *BRCA* status includes germline or tumor pathogenic mutations in *BRCA1* or *BRCA2;* *IQR* Interquartile range, *p*-value form Fisher’s exact test for categorical variables or Wilcoxon rank sum test for numeric variables.

Among patients with multiple mutations, analysis of the 5 most frequently mutated genes (see Supplementary Methods) revealed significant co-occurrence of *PPM1D* and *TP53* mutations (OR 8.2, FDR-adjusted *p* = 0.046; Fisher’s exact test; Fig. 1f).

### Associations of clonal hematopoiesis with clinical characteristics and outcome measures

**Fig. 2:**
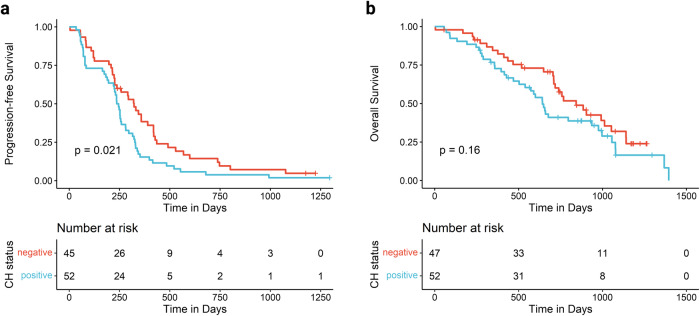
Survival analysis of the 103 patients enrolled in the EUDARIO study. **a** Kaplan-Meier analysis of progression-free survival stratified by CH status. **b** Kaplan-Meier analysis of overall survival stratified by CH status.

We next assessed the association of CH with clinical characteristics, laboratory measures, occurrence of therapy-related complications, and survival. A total of 53 patients (51%) had at least one somatic mutation in a typical CH gene (Supplementary Table S2), with a VAF ≥ 1%. The demographic and clinical characteristics of the patients at baseline are shown in Table 1. Patients with CH were significantly older than those without CH (median 68 vs 55 years, *p* < 0.001) and had lower levels of hemoglobin (12.3 vs 13.0 g/dl, *p* = 0.004, Table 1) at initiation of treatment. In terms of therapy-related complications, patients with CH had higher rates of infectious complications (49% *vs* 26%, *p* = 0.025; Supplementary Table S5a). No significant differences between occurrences of cytopenias or hematotoxicity-related treatment interruptions were noted during carboplatin or maintenance treatment between patients with and without CH (Supplementary Tables S5b, c and S6). No t-MN development was reported during the median follow-up period of 22 months in the EUDARIO trial. The presence of CH was not associated with best response to treatment (Supplementary Table S7). CH-positive patients had shorter PFS (median PFS 7.9 months *vs* 10.6 months, *p* = 0.021 in log-rank test), and a trend for shorter OS (median OS 21.1 months *vs* 27.7 months, *p* = 0.16) in univariate survival analysis (Fig. 2). In a multivariate Cox regression model with age, treatment arm, number of previous therapy lines, and prior PARPi treatment as covariates, prior PARPi therapy was the only variable associated with shorter PFS (hazard ratio 2.55, 95%-CI 1.35–4.80, *p* = 0.004) and shorter OS (hazard ratio 2.33, 95%-CI 1.06–5.10, *p* = 0.03; Supplementary Fig. S1a, b).

### Detection of clonal hematopoiesis in cell-free DNA

**Fig. 3:**
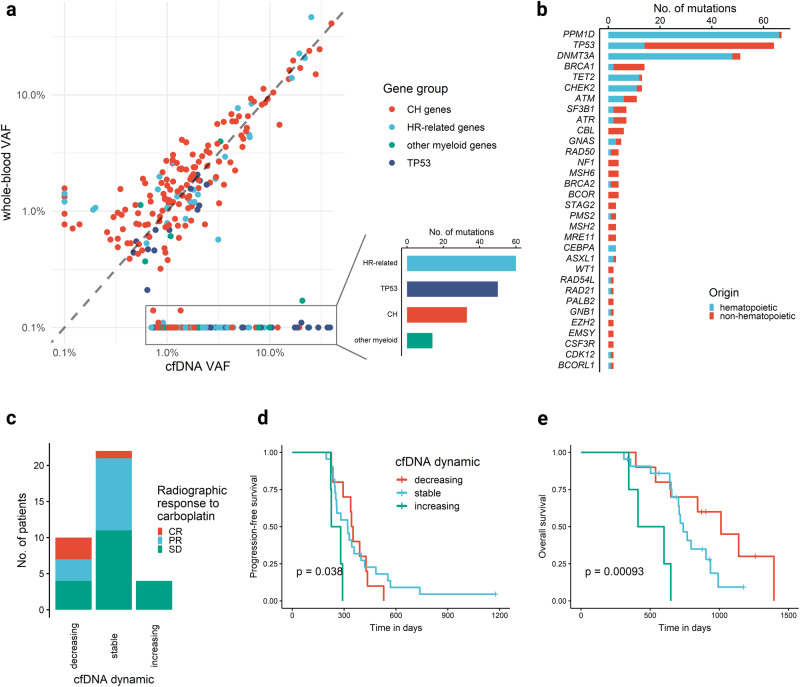
Detection of clonal hematopoiesis in cell-free DNA. Mutational landscape of 340 somatic mutations detected in cfDNA of 102 patients from the EUDARIO study. **a** VAFs of somatic mutations detected in cfDNA and/or WB DNA at initiation of study treatment. VAFs of mutations detected in only one DNA source were set to the detection limit of 0.1% in the other for the purpose of visualization on a logarithmic scale. Color depicts different mutated gene groups. *TP53* mutations are shown as a separate group due to their abundance. **b** Prevalence of somatic mutations detected in cfDNA by mutated genes. Color depicts hematopoietic *vs* non-hematopoietic origin depending on the VAF ratio between WB and cfDNA. **c** Radiographic response to carboplatin treatment in 36 HGOC patients stratified by the VAF dynamics of *TP53* mutations of non-hematopoietic origin in cfDNA during carboplatin treatment. **d** Progression-free survival stratified by the *TP53* VAF dynamics. **e** Overall survival stratified by *TP53* VAF dynamics.

Next, we explored the landscape of somatic mutations in cfDNA extracted from plasma samples. A corresponding plasma sample at the initiation of study treatment was available for 102 patients. A total of 340 somatic mutations were identified in cfDNA and all but 5 mutations with VAF ≥ 1% detected in WB samples were also detectable in cfDNA (Fig. 3a), with a strong correlation of VAFs (Pearson correlation coefficient R = 0.83, *p* < 10^−15^, Supplementary Fig. S2), implying that the clone sizes (as measured by the VAF) of CH clones can also be quantified in cfDNA with reasonable accuracy. Depending on the VAF ratio between WB and cfDNA, 157 of the 340 mutations were classified as non-hematopoietic in origin (Fig. 3a, b), indicating that these mutations were most likely tumor-derived. The mutation composition differed significantly between the 2 compartments: mutations that were classified as non-hematopoietic had a high fraction of *TP53* mutations (the most frequently mutated gene in HGOC) and mutations in HR-related genes, while the most frequently affected CH genes *DNMT3A* (2/157), *TET2* (1/157), *ASXL1* (1/157) and *PPM1D (*1/157*)* were rarely mutated (Fig. 3a, b). Notably, a substantial proportion of cfDNA mutations detected in genes commonly mutated in HGOC, specifically *TP53* (14/64 = 18%) and HR-related genes (as defined in Supplementary Table S2; 28/88 = 32%), were of hematopoietic origin.

To corroborate the tumor-origin of *TP53* mutations classified as non-hematopoietic in origin, we categorized their VAF dynamics in cfDNA during carboplatin treatment as increasing (VAF increase by factor 10), decreasing (VAF decrease by factor 10), or stable (all others), which correlated with radiographic response to carboplatin, PFS, and OS (Fig. 3c, d). In conclusion, these observations underline the importance of parallel sequencing of WB and cfDNA in liquid biopsies to correctly attribute the origin of cfDNA mutations.

### Clonal dynamics under DNA-damaging therapy

**Fig. 4:**
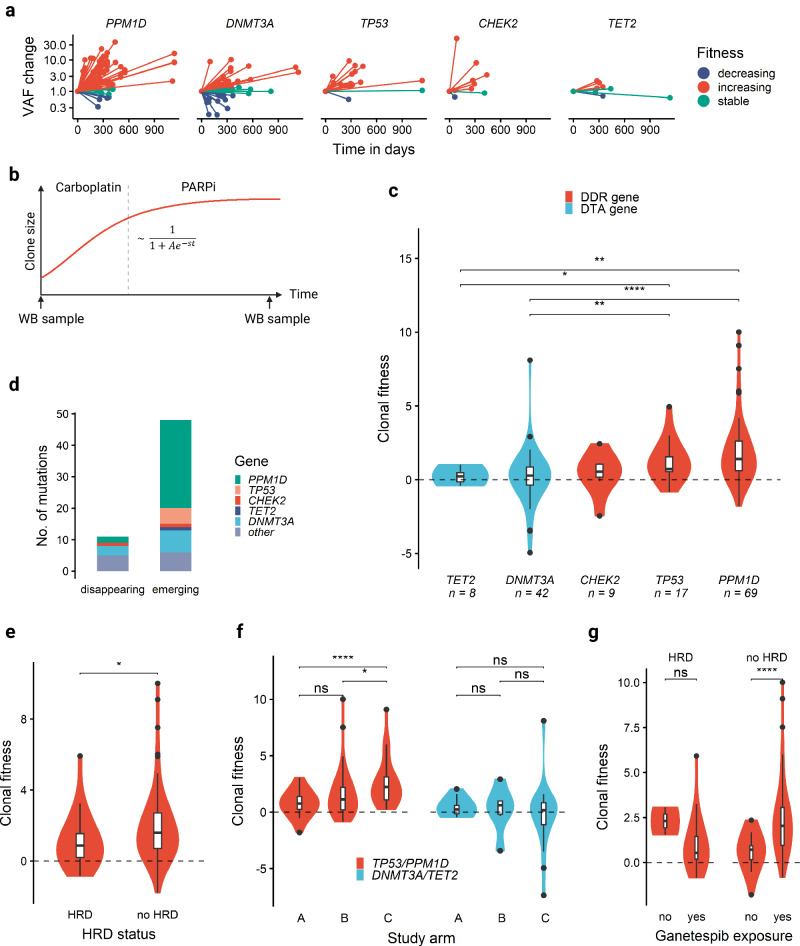
Clonal dynamics under DNA-damaging therapy. Clonal fitness measured in paired WB samples taken at the beginning and end of study treatment for each patient (*n* = 61 patients). **a** Relative VAF changes over time between initiation and end of treatment. Color denotes the category of clonal fitness, defined as increasing if clonal fitness *s* > 0.25/year, decreasing if *s* < −0.25/year and stable otherwise. **b** Schematic model for the evolution of clone size as a sigmoid function over time over the whole treatment course (no differentiation between chemotherapy and maintenance phase). **c** Violin plot of clonal fitness per gene for the 5 most frequently mutated genes. DTA genes are colored in blue, DDR genes in red. **d** Number of increasing/decreasing clones passing the 1% VAF threshold colored by mutated gene/gene group. **e** Median clonal fitness of *TP53-* and *PPM1D-* mutated clones in patients with and without germline HRD mutations. **f** Median clonal fitness of *DNMT3A*/*TET2-* and *TP53/PPM1D*-mutated clones in different treatment arms with increasing exposure to HSP90 inhibition (arm A – no HSP90i, arm B – HSP90i during carboplatin treatment, arm C – HSP90i during carboplatin and PARPi treatment. **g** Median clonal fitness of *DNMT3A*/*TET2-* and *TP53/PPM1D*-mutated clones in patients with and without germline HRD mutations. Asterisks denote level of statistical significance: * < 0.05, ** < 0.01, *** < 0.001, **** < 0.0001.

To investigate the dynamics of hematopoietic clone size under carboplatin, PARP inhibition and HSP90 inhibition, we first analyzed paired WB samples from each patient obtained at the beginning and end of the study treatment (*n* = 61 patients). The relative VAF changes in *PPM1D, DNMT3A, TP53, CHEK2* and *TET2* mutations, categorized as increasing, stable, or decreasing, are shown in Fig. 4a. We found expansion of *TP53*-mutated clones in 25% (15/61) and *PPM1D*-mutated clones in 43% (26/61) of patients during the study period. For each mutation, we estimated the clonal fitness *s* as previously described [41, 49] by modeling clonal growth as a sigmoid function over time (Fig. 4b). Gene-wise comparisons revealed that *PPM1D*- and *TP53*-mutated clones had significantly higher median fitness than *TET2*- or *DNMT3A*-mutated clones (Fig. 4c). Of note, 48 clones that initially had a VAF < 1% emerged during treatment, including 28 *PPM1D* and 5 *TP53* mutations. 11 mutations, on the other hand, fell below the 1% VAF threshold during treatment (Fig. 4d). Interestingly, the median fitness in *TP53-* and *PPM1D-*mutated hematopoietic clones was significantly lower in patients with germline HRD (Supplementary Table S3b), than in those without germline HRD (0.89/year *vs* 1.59/year, *p* = 0.04, Fig. 4e). While time on treatment was identical between patients with and without germline HRD (data not shown), episodes of thrombocytopenia occurred more frequently in patients with germline HRD (Supplementary Tables S8a, b), which however did not lead to significantly more treatment interruptions or discontinuations (Supplementary Tables S9a, b).

To delineate the influence of HSP90 inhibition, we leveraged the randomized design of the study into 3 arms with increasing HSP90i exposure (none in arm A, arm B < arm C). While the median clonal fitness of *DNTM3A-* and *TET2-*mutated clones did not differ among the 3 arms, we observed a significant increase in clonal fitness in *PPM1D*- and *TP53-*mutated clones with increasing HSP90i exposure (Fig. 4f). However, Ganetespib exposure correlated with an increase in clonal fitness of *TP53/PPM1D*-mutated clones only in the absence of germline HRD (Fig. 4g).

**Fig. 5:**
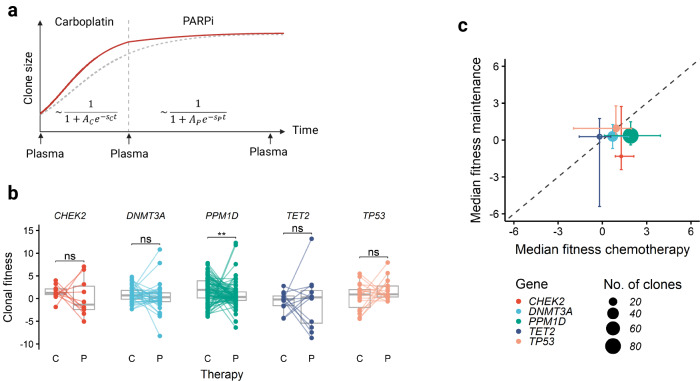
Comparison of clonal fitness under carboplatin and PARPi therapy. **a** Schematic model of evolution of clone size (estimated from cfDNA VAF) as a sigmoid function over time for carboplatin and PARPi maintenance treatment separately. **b** Comparison of individual clonal fitness estimates between carboplatin (C) and PARPi maintenance (P) for the 5 most frequently mutated genes. **c** Scatterplot showing median fitness estimates for the 5 most frequently mutated genes during chemotherapy (x-axis) and PARPi maintenance (y-axis). Error bars denote the interquartile range. Point size reflects the number of clones with mutations in the respective gene. Asterisks denote level of statistical significance: * < 0.05, ** < 0.01, *** < 0.001, **** < 0.0001.

Next, we aimed to differentiate clonal fitness between carboplatin *versus* PARPi maintenance treatment. To overcome the lack of WB samples at the initiation of Niraparib maintenance, we used cfDNA to estimate CH clone size at 3 timepoints: initiation of study treatment, initiation of maintenance therapy and end of study treatment (Fig. 5a) in 49 patients (median duration of chemotherapy 5.8 months, range 4.9–8.2 months; median duration of maintenance 5.5 months, range 1.2–31.4 months). In many cases, clonal fitness during carboplatin treatment diverged from clonal fitness during PARPi maintenance treatment (Fig. 5b). Interestingly, median fitness of *PPM1D*-mutant clones was significantly lower during PARPi treatment than during carboplatin treatment (median 0.36/year *vs* 1.93/year, *p* = 0.003 in paired Wilcoxon rank sum test), which was not the case for *TP53*-mutated clones (Fig. 5c).

### Clonal architecture of therapy-related clonal hematopoiesis with multiple co-occurring DDR mutations

Finally, the high abundance of co-occurring DDR mutations in this patient collective led us to investigate the clonal architecture of therapy-related clonal hematopoiesis at single-cell resolution. WB specimens from an additional set of 37 HGOC patients undergoing platinum- and/or PARPi treatment (see Supplementary Table S10 for demographic and clinical characteristics) were screened for the presence of CH using the same sequencing pipeline. 56 somatic mutations with VAF ≥ 1% were identified in 30 patients, with *DNMT3A*, *PPM1D*, and *TP53* being the most frequently mutated genes (Supplementary Figure S3). In line with our findings, DDR gene mutations were closely associated with the number of prior therapy lines (81% DDR-mutated with > 1 therapy line *vs* 33% in DDR-wildtype, *p* = 0.007) and the duration of prior PARPi treatment (median 18.5 months in DDR-mutated *vs* 9 months in DDR-wildtype, *p* = 0.033). Among 30 CH-positive patients, 14 had multiple mutations and 5 had co-occurring mutations in DDR genes.

**Fig. 6:**
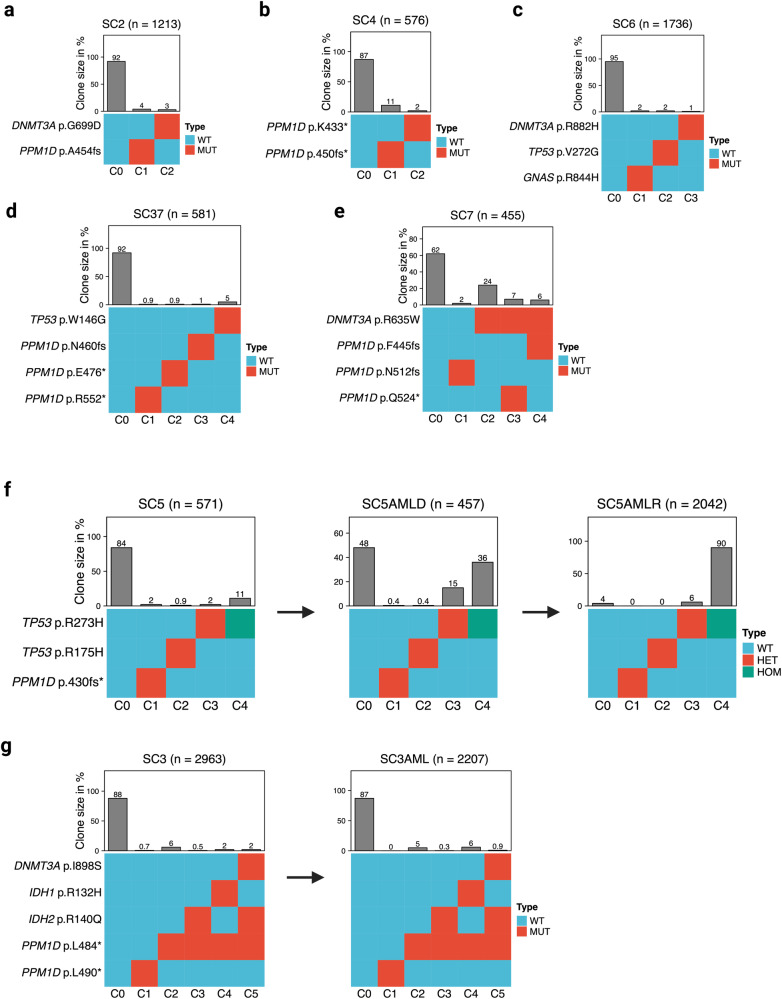
Clonal architecture of therapy-related CH mutations in selected cases at single-cell resolution. Single cell sequencing analysis of 7/37 prospectively collected patient samples with multiple CH mutations (pseudonymized SC1 – SC37). Heatmaps visualize the clonal architecture with somatic mutations identified from bulk sequencing on the y-axis and inferred clones (labeled C1, C2, C3, …) on the x-axis (C0 represents the collection of all cells not carrying any of the listed mutations). Bar charts depict the abundance of their corresponding mutant clones as derived from single-cell sequencing data, n denotes the number of cells genotyped. Mutations are heterozygous if not stated otherwise. **a**–**e** Cases with multiple CH mutations. **f** Progression to t-AML at single-cell resolution in case 1. At the time of first sampling this patient was 66 years and on olaparib maintenance treatment for her first relapse of metastatic OC at the time of sampling. She had received carboplatin/paclitaxel/bevacizumab at first diagnosis and carboplatin/pegylated liposomal doxorubicin at relapse. Five months after blood sampling she was started on aromatase inhibitor treatment for her second relapse. She developed progressive pancytopenia and was diagnosed with therapy-related myeloid dysplastic syndrome 6 months after blood sampling, which rapidly progressed to AML. The AML was refractory to induction with CPX351 (sample SC5-AMLD) and was then treated with venetoclax/5-azacitidine for 6 cycles until progression (sample SC5-AMLR). **g** Progression to t-AML at single-cell resolution in case 2. This patient was a 52-year-old woman who at the time of first sampling (SC-3) was on olaparib maintenance treatment for her first relapse of OC. Treatment at initial diagnosis was carboplatin and bevacizumab. At first relapse she received re-induction with carboplatin/pegylated liposomal doxorubicin resulting in a CR, followed by olaparib maintenance for 4.5 years. She developed persistent cytopenia and was diagnosed with t-AML (SC3-AML) 5 months after first sampling. After 4 cycles of venetoclax/5-azacitidine, she achieved CR and proceeded to allogeneic stem cell transplantation from an HLA-identical unrelated donor.

Eleven samples from 7 patients (Supplementary Table S11) were selected for single-cell DNA sequencing based on their co-mutational pattern, with a focus on DDR mutations. In all cases with multiple DDR mutations (14 DDR mutation pairs in 5 patients), each mutation resided in a separate clone (Fig. 6a–g). Two of 37 patients (5%) developed therapy-related acute myeloid leukemia (t-AML) within 6 months of follow-up (Fig. 6f, g). Case SC5 carried a *TP53*-mutant clone with loss of heterozygosity, which had substantially expanded at the time of AML diagnosis and expanded further at the time of relapse after treatment with venetoclax and 5-azacitidine, whereas the other clones had disappeared (Fig. 6f). Case SC3 had a large *PPM1D*-mutated clone at the time of first sampling, with 2 independent *IDH1*- and *IDH2*-mutant subclones. At the time of AML diagnosis, the *PPM1D*/*IDH1*-mutant subclone had expanded (Fig. 6g). We conclude that in patients with multiple DDR-mutant clones there is mutual clonal exclusivity for individual DDR mutations in hematopoietic stem cells. Moreover, DDR-mutant clones were the origin of t-AML in both investigated cases.

## Discussion

In the present study, we performed a detailed analysis of the prevalence, mutational spectrum, and clonal architecture of CH in patients with relapsed HGOC and quantified clonal fitness under serial treatment with carboplatin and PARPi in combination with HSP90 inhibition. Our data revealed a high overall prevalence of CH and specifically of DDR-CH at study entry in this patient cohort (57% and 35% of patients, respectively), and provide compelling evidence that in addition to the known association with prior platinum treatment [12], PARPi and HSP90i therapy independently pose an increased risk for the expansion of high-fitness DDR-CH in a dose-dependent manner.

While large studies of the longitudinal history of CH over the patient’s life span reported a slow growth rate of *TP53* and *PPM1D* mutations in the absence of external stressors [49, 53, 54], our clonal fitness analysis points towards a strong selection of DDR-mutated clones over the course of study treatment. We observed an increase in clonal fitness of DDR-CH in response to increasing cytotoxic stress across the 3 treatment arms with increasing exposure to HSP90 inhibition. Interestingly, this selective advantage was partially abrogated by the presence of germline HRD mutations, however, the present study cannot fully delineate factors contributing to this association. While the median clonal fitness of *TP53*-mutated clones during PARPi maintenance was identical to that during carboplatin treatment, the clonal fitness of *PPM1D*-mutated clones was significantly lower during PARPi maintenance. Although no t-MN development was reported in the EUDARIO trial, likely due to the aggressive nature of the underlying disease and a short median overall survival of 24 months, we demonstrated the expansion of *TP53*-mutated clones in 25% (15/61) and *PPM1D*-mutated clones in 43% (26/61) of patients during treatment. While the risk for progression to t-MN is best documented for *TP53* mutations [21, 34, 55, 56] both *TP53*- and *PPM1D*-driven CH have recently been associated with cardiovascular complications [41, 57, 58]. A recent case series of patients with OC under PARPi treatment who developed persistent cytopenia reported that in some cases cytopenia and clonal abnormalities reversed upon discontinuation of PARPi treatment, providing a rationale for close monitoring of certain high-risk patients [59].

Single-cell level analysis to elucidate the clonal architecture in patients harboring multiple DDR mutations showed that each mutation emerged in a separate clone. *PPM1D* mutations are truncating variants that predominantly occur in exon 6 and are considered to be gain-of-function mutations that impair wild-type *TP53* function [13, 14], phenocopying the dominant-negative effect of missense *TP53* mutations [16]. Accordingly, this observation is likely a consequence of the strong selective pressure from cytotoxic treatment, leading to positive selection of multiple clones with similar traits that converge on resistance to DNA damage-induced apoptosis. As evidenced by single-cell genotyping, large DDR-clones were indeed the origin of subsequent t-AML that developed within 6 months after the initial sampling in both cases investigated.

Finally, our data enabled a comparison of the landscapes of somatic mutations in cfDNA and WB DNA. Liquid biopsies are emerging as non-invasive diagnostic tools that have potential applications in non-invasive tumor profiling as well as in the prediction and evaluation of therapy response [60–63]. For patients with metastatic castration-resistant prostate cancer, cfDNA testing to detect HRD mutations as an eligibility criterion for olaparib treatment has already been integrated into the clinical routine [64]. Parallel sequencing of cfDNA and WB DNA in our patient cohort revealed significant overlap in the mutational profiles derived from hematopoietic and non-hematopoietic cells in cfDNA, specifically affecting *TP53* and HR-related mutations. These findings underscore the potential confounding impact of CH mutations on the interpretation of tumor-derived mutations in liquid biopsies, when solely relying on the detection of the latter in cfDNA without concurrent sequencing of tumor material or WB, as was previously shown in prostate cancer patients [65].

In conclusion, this study provides unique insights into the architecture and the preferential selection of DDR-mutated hematopoietic clones under intense DNA-damaging treatment. Specifically, PARPi and HSP90i therapy independently pose an increased risk for the development of DDR-CH in a dose-dependent manner.

### Supplementary information

Arends et al_supplemental information

Arends et al_Supplementary Table S3

## Data Availability

The datasets used and/or analysed during the current study are available from the corresponding author on reasonable request. All codes are available on the GitHub repository https://github.com/arendscm/CH_EUDARIO.git.
