# Correction: Dynamics of clonal hematopoiesis under DNA-damaging treatment in patients with ovarian cancer

**DOI:** 10.1038/s41375-024-02274-y

**Published:** 2024-05-07

**Authors:** Christopher Maximilian Arends, Klara Kopp, Raphael Hablesreiter, Natalia Estrada, Friederike Christen, Ute Martha Moll, Robert Zeillinger, Wolfgang Daniel Schmitt, Jalid Sehouli, Hagen Kulbe, Maximilian Fleischmann, Isabelle Ray-Coquard, Alain Zeimet, Francesco Raspagliesi, Claudio Zamagni, Ignace Vergote, Domenica Lorusso, Nicole Concin, Lars Bullinger, Elena Ioana Braicu, Frederik Damm

**Affiliations:** 1 Department of Hematology, Oncology, and Cancer Immunology, Charité–Universitätsmedizin Berlin, corporate member of Freie Universität Berlin and Humboldt-Universität zu Berlin, Berlin, Germany; 2 Department of Pathology, Stony Brook University Cancer Center, Stony Brook, NY 11794 USA; 3 Department of Obstetrics and Gynaecology, Molecular Oncology Group, Comprehensive Cancer Center-Gynaecologic Cancer Unit, Medical University of Vienna, Vienna, Austria; 4 Department of Pathology, Charité - Universitätsmedizin Berlin, corporate member of Freie Universität Berlin and Humboldt Universität zu Berlin, Berlin, Germany; 5 Department of Gynaecology, European Competence Center for Ovarian Cancer, Charité-Universitätsmedizin Berlin, Corporate Member of Freie Universität Berlin, Humboldt-Universität zu Berlin, and Berlin Institute of Health, Campus Virchow Klinikum, Berlin, Germany; 6 North Eastern German Society for Gynecological Cancer. Tumor Bank Ovarian Cancer Network, Berlin, Germany; 7 Klinik für Innere Medizin II, Abteilung Hämatologie und Onkologie, Universitätsklinikum Jena, Jena, Germany; 8 Centre Anticancereux Léon Bérard, University Claude Bernard Lyon, GINECO Group, Lyon, France; 9 Department of Obstetrics and Gynecology, Medical University of Innsbruck, Austrian AGO, Innsbruck, Austria; 10 Gynecologic Oncology Unit Fondazione IRCCS Istituto Nazionale Tumori, Milan, Italy; 11 Division of Oncology, IRCCS Azienda Ospedaliero-Universitaria di Bologna, Bologna, Italy; 12 Division of Gynecological Oncology, Department of Gynecology and Obstetrics, Leuven Cancer Institute, Katholieke Universiteit Leuven, Leuven, Belgium; 13 Belgium and Luxembourg Gynaecological Oncology Group (BGOG), Leuven, Belgium; 14 Humanitas San Pio X, Humanitas University Rozzano, Milan, Italy; 15 German Cancer Consortium (DKTK) and German Cancer Research Center (DKFZ), Heidelberg, Germany

**Keywords:** Stem-cell research, Cancer genomics, Cancer genetics

Correction to: *Leukemia* 10.1038/s41375-024-02253-3, published online 18 April 2024

In the version of the article initially published, important funding information was missing. ‘This study was partially funded by EU FP7-HEALTH - Specific Programme “Cooperation”: Health (Grant agreement ID: 602602). UM was supported by National Cancer Institute 2R01CA176647 and by a Carol Baldwin Foundation TRO’ has been added.

The original article has been corrected.”

