# Supplementary material for: Dynamics of clonal hematopoiesis under DNA-damaging treatment in patients with ovarian cancer
Source: Leukemia. 2024 Apr 18;38(6):1378–89. doi: 10.1038/s41375-024-02253-3 (PMC11147769; doi:10.1038/s41375-024-02253-3)
Supplement: Supplementary file 1 — Arends et al_supplemental information [file 41375_2024_2253_MOESM1_ESM.docx]

# Supplementary Methods

## Patients

The European Trial on Enhanced DNA Repair Inhibition in Ovarian Cancer (ENGOT-ov48/EUDARIO) study (NCT03783949) is a multicenter, randomized, open-label, phase ll trial performed in women with relapsed, platinum-sensitive HGOC. By definition, patients had experienced progressive disease > 6 months after previous platinum-based treatment. A total of 120 women of age older than 18 years with a histologically proven diagnosis of high-grade serous, high-grade endometrioid, undifferentiated epithelial ovarian cancer, carcinosarcoma, fallopian tube or primary peritoneal cancer, were randomized 1:1:1 to three different treatment arms. Stratification criteria included germline or somatic *BRCA* mutation, prior lines of chemotherapy and prior PARPi treatment. Study participants were treated at seven institutions in Europe (Berlin, Innsbruck, Leuven, Lyon, Mailand, Bologna and Rome) between 30th January 2019 and 22nd December 2022. The backbone of all treatment arms consisted of 6 cycles of carboplatin-based chemotherapy followed by maintenance therapy with Niraparib. In the two experimental arms, the study drug Ganetespib, a heat-shock protein 90 (HSP90) inhibitor^1^, was administered during chemotherapy (arm B) or during chemotherapy and maintenance (arm C; see Supplementary Table S1 for a detailed treatment plan). The primary endpoint of the ENGOT-ov48/EUDARIO study was progression-free survival (PFS), secondary outcome measures included overall survival (OS), adverse events and best response. For single-cell sequencing analysis, further 37 patients with OC receiving PARPi and/or platinum treatment were prospectively recruited at the Gynecology outpatient department of Charité Campus Virchow Klinikum, Berlin, Germany. Written consent was obtained from all patients in accordance with the Declaration of Helsinki and the study was approved by local ethics committees.

## Materials

Peripheral whole blood (WB) and plasma specimen of ENGOT-ov48/EUDARIO participants were collected at the initiation of treatment and at predefined timepoints along the treatment course. WB and plasma samples collected in the ENGOT-ov48/EUDARIO study were stored in liquid nitrogen tanks until further processing. Genomic DNA from WB samples was extracted using the QIAmp DNA Mini Kit (QIAGEN, Venlo, Netherlands) according to the manufacturer’s protocol. CfDNA was extracted from 1 ml plasma using the MagMAX Cell-free DNA Isolation Kit (ThermoFisher Scientific, Waltham, MA, USA) according to the manufacturer’s protocol. Peripheral WB samples from the additional 37 patients and three bone marrow (BM) samples from two patients who consecutively developed t-AML were processed with Ficoll density centrifugation as previously described^2^ and viable mononuclear cells were stored in liquid nitrogen until further use.

## Sequencing analysis

The sequencing data was processed using our in-house snakemake^3^ pipeline, following previously described methods.^4,5^ UMIs were extracted, and FASTQ files were generated using picard ExtractIlluminaBarcodes, IlluminaBasecallsToSam, and SamToFastq (Picard Tools, Broad Institute). The raw reads were aligned to the GRCh38 reference genome using bwa mem^6^, and UMI information was added using picard MergeBamAlignment. Consensus reads were generated using fgbio GroupReadsByUmi with the "-s adjacency" option and fgbio CallMolecularConsensusReads with "-M 3" (https://github.com/fulcrumgenomics/fgbio). These consensus reads were then aligned to GRCh38 using bwa mem and picard MergeBamAlignment. Quality filtering of the aligned consensus reads was performed using fgbio FilterConsensusReadsQuality with a minimum of 3 consensus reads and default parameters. Local realignment was conducted using GATK3 RealignerTargetCreator and IndelRealigner.^7,8^ Variant calling was performed using VarDict in single-mode with a minimum allele frequency of 0.001.^9^ Subsequently, variant calls were annotated using ANNOVAR^10^ with public databases, including RefSeq^11^, gnomAD30_genome^12^, avsnp150^13^, and cosmic92_coding^14^.

## Variant filtering

The list of variants was further filtered and processed as follows: nonsynonymous variants in the coding region and splice site variants with a minimum alternate allele count of 10 consensus reads, population-based allele frequency (AF) in the gnomAD database < 0.01, that were called in < 20% of samples on the same sequencing lane and had a binomial approximation score < -12 were retained. The binomial approximation score represents a measure of how likely a variant call with alternate allele count *a* at a position would emerge from background noise, and was defined as

$$\log_{10} (1-P_{N,p}(X\leq a)),$$

where *P_N,p_(X ≤ a)* is the cumulative binomial distribution with *N* = read depth, *p* = median VAF over the sequencing lane at the respective position, and *a* is the alternate allele count of the variant. All variants with VAF > 45% were classified as germline mutations (if the VAF was not < 45% at any other available timepoint). Furthermore, variants with VAF > 40% that were reported in the dbSNP database as single nucleotide polymorphisms (SNPs) or had a population-based allele frequency (AF) > 1% in the gnomAD database, were classified as SNPs. Highly recurrent single nucleotide variants (*DNMT3A* codon R882, *GNB1* K57E, *JAK2* V617F, *SF3B1* codon K666 and K700, *SFRS2* codon P95, and *U2AF1* codon S34 and Q157) were retained. All somatic mutation candidates passing these filters were manually revised in the Integrative Genomics Viewer.^15^

For the analysis of paired WB and cfDNA samples, variants that were detected in either source with a VAF ≥ 0.8%, or in both sources with sum of the two VAFs ≥ 0.8% were used for further analysis. Variants in cfDNA were classified as of non-hematopoietic origin, if the VAF in cfDNA was five-fold higher than in WB DNA. In the serial sample analysis in WB DNA and cfDNA, variants that were detected in at least two timepoints with VAF > 0.8% in at least one timepoint were retained. For variants not detected in all available timepoints, sequencing data was manually reviewed for the presence of the variant beneath the variant calling threshold (0.1%), and, if present, the VAF was manually set to the detection threshold of 0.1%.

## Co-mutation analysis

Gene interaction analysis to determine significant co-occurrence / exclusivity in multiply mutated patients was carried out for the five most frequently mutated genes as described by Gerstung *et al*.^16^, using the somaticInteractions()function of the maftools R package for the top five genes.

**Single-cell sequencing**

Single-cell DNA sequencing was performed on the MissionBio Tapestri platform using the Tapestri Single-Cell DNA Sequencing V2 kit (MissionBio, South San Francisco, CA, USA). In some cases, a sample-multiplexing approach was applied, in which the individual SNP profile was used as specific sample marker, enabling sample pooling and bioinformatic decoding. Two samples with at least five distinct SNP positions, i.e. wild-type compared to homozygous or heterozygous, were paired, and cells from both patients mixed and processed as one library. After sequencing, each barcoded cell was assigned to one of the two patients according to the SNP profile.

For library preparation, 1 ml of frozen BM or PB mononuclear cells were thawed in a pre-warmed complete growth medium (10% FBS in IMDM). Cells were counted with the Countess 3 Automated Cell Counter (Invitrogen, Waltham, MA, USA). If cell viability was <80%, a dead-cell removal step was applied to the sample using EasySep™ Dead Cell Removal (Annexin V) kit (StemCell Technologies, Vancouver, BC, Canada). Afterwards, the cells were diluted to a concentration of 3,000 to 4,000 cells/µl in cell buffer. A total of 35 µl containing on average 122,500 cells from two multiplexed samples were loaded on the Tapestri platform for cell encapsulation and cell barcoding.

Library construction was performed according to the Tapestri Single-Cell DNA Sequencing V2 manufacturer's protocol and using the MissionBio Myeloid or Myeloid Koichi Takahashi (MDACC)^17^ panels, depending on best amplicon coverage of the previously identified patient's somatic mutations.

The libraries were pooled to a concentration of 5 mM and sequenced on the NovaSeq 6000 platform (Illumina, San Diego, CA, USA), using the S1 flow-cell for a 150 bp paired-end run with a 15% ratio of PhiX DNA.

## Single-cell sequencing bioinformatic workflow

Sequencing reads were processed using Mission Bio’s Tapestri pipeline v2.0.2 with default parameters. An adapted preprocessing script from COMPASS^18^ in combination with a whitelist of known SNPs and somatic mutations was used for each sample pair to retrieve the number of reads at each locus supporting the mutated or wildtype alleles. Here default parameters with a minimum read depth of 6 and a minimum VAF of 0.2 for heterozygous variants were used. Each variant in each cell is annotated as one of four different states: (0) wildtype, (1) heterozygous, (2) homozygous or (3) missing. BnpC^19^ was used to cluster the cells into different genotypes based on their reported SNPs with 10 Markov chain Monte Carlo (MCMC) chains with 25,000 steps in parallel, “-pp 0.75 0.75” as argument to beta distribution and default parameters. Prior a quality filtering step was performed removing cells with more than one missing SNP in case of up to five SNPs in total or with more than two SNPs in case of more than five SNPs in total. Clusters were assigned to the corresponding sample based on their SNP profile. Downstream analysis was performed when at least 400 cells remained for a sample.

Clones in each sample were identified by converting each somatic variant in each cell in one of the following categories: (0) wildtype, (1) mutated or (3) missing. A threshold of at least five cells to report a clone was used. A snakemake^3^ pipeline for this deconvolution was established. The data handling was performed in R (R Foundation for Statistical Computing, Vienna, Austria) version 4.2.1 with the libraries ggplot2, complexHeatmap, stringr, dplyr, tidyr.

# Supplementary Tables

## Supplementary Table S1

Table S1: Detailed dosage information of therapy according to treatment arm

| Arm | Treatment | |
| --- | --- | --- |
| A (Standard) |  | Carboplatin (AUC5 d1, q3w i.v.)  Paclitaxel (175 mg/m² d1, q3w i.v.)  or  Carboplatin (AUC4 d1, q3w i.v.)  Gemcitabine (1000 mg/m² d1, d8, q3w i.v.) |
|  | Maintenance: | Niraparib (200 - 300 mg oral daily, q4w) |
| B (First experimental) |  | Carboplatin (AUC5 d1, q3w i.v.)  Ganetespib (150 mg/m2, d1, q3w) |
|  | Maintenance: | Niraparib (200 - 300 mg oral daily, q4w) |
| C (Second experimental) |  | Carboplatin (AUC5 d1, q3w i.v.)  Ganetespib (150 mg/m² d1, q3w i.v.) |
|  | Maintenance: | Niraparib (200 mg oral daily, q4w)  Ganetespib (100 mg/m² d1, d8, d15, d22, q4w i.v.) |

## Supplementary Table S2

Table S2: Genes covered by the custom panel used in this study, grouped into typical CH genes, other myeloid genes, and HR-related genes.

| No. | CH genes | Region |  | No. | | Other myeloid genes | Region |  | No. | HR-related genes | Region |
| --- | --- | --- | --- | --- | --- | --- | --- | --- | --- | --- | --- |
| 1 | *DNMT3A* | Full |  | | 1 | *KRAS* | Full |  | 1 | *BRCA1* | Full |
| 2 | *TET2* | Full |  | | 2 | *NRAS* | Full |  | 2 | *BRCA2* | Full |
| 3 | *JAK2* | Full |  | | 3 | *EZH2* | Full |  | 3 | *EMSY* | Full |
| 4 | *ASXL1* | Full |  | | 4 | *MYD88* | Full |  | 4 | *PTEN* | Full |
| 5 | *SF3B1* | Full |  | | 5 | *CSF3R* | E14,17 |  | 5 | *RAD51* | Full |
| 6 | *SRSF2* | Full |  | | 6 | *ETV6* | Full |  | 6 | *RAD51C* | Full |
| 7 | *TP53* | Full |  | | 7 | *FLT3* | E6,14,15,20 |  | 7 | *RAD51D* | Full |
| 8 | *U2AF1* | Full |  | | 8 | *GATA2* | Full |  | 8 | *RAD50* | Full |
| 9 | *PPM1D* | Full |  | | 9 | *GATA1* | E2 |  | 9 | *RAD52* | Full |
| 10 | *CBL* | Full |  | | 10 | *KIT* | E8-11,17 |  | 10 | *RAD54L* | Full |
| 11 | *IDH1* | Full |  | | 11 | *MPL* | E10 |  | 11 | *CDK12* | Full |
| 12 | *IDH2* | Full |  | | 12 | *NPM1* | E11 |  | 12 | *ATR* | Full |
| 13 | *BCOR* | Full |  | | 13 | *RUNX1* | Full |  | 13 | *BARD1* | Full |
| 14 | *BCORL1* | Full |  | | 14 | *SETBP1* | E4-9 |  | 14 | *BRIP1* | Full |
| 15 | *RAD21******** | Full |  | | 15 | *NF1* | E28-38 |  | 15 | *CHEK1* | Full |
| 16 | *STAG2* | Full |  | | 16 | *PHF6* | E3,4,5,7,8,9 |  | 16 | *FAM175A* | Full |
| 17 | *CHEK2******** | Full |  | | 17 | *BRAF* | E15 |  | 17 | *NBN* | Full |
| 18 | *GNAS* | Full |  | | 18 | *NOTCH1* | E26,27,34 |  | 18 | *PALB2* | Full |
| 19 | *GNB1* | Full |  | | 19 | *XPO1* | E14 |  | 19 | *MRE11* | Full |
| 20 | *ATM******** | Full |  | | 20 | *CALR* | E8-9 |  | 20 | *MLH1* | Full |
| 21 | *WT1* | Full |  | | 21 | *CEBPA* | Full |  | 21 | *MSH2* | Full |
| 22 | *PTPN11* | Full |  | |  |  |  |  | 22 | *MSH6* | Full |
| 23 | *STAT3* | Full |  | |  |  |  |  | 23 | *PMS2* | Full |
| 24 | *BRCC3******** | Full |  | |  |  |  |  | 24 | *FANCA* | Full |
|  |  |  |  | |  |  |  |  | 25 | *FANCC* | Full |
|  |  |  |  | |  |  |  |  | 26 | *FANCI* | Full |
|  |  |  |  | |  |  |  |  | 27 | *FANCL* | Full |
|  |  |  |  | |  |  |  |  |  |  |  |

* also considered as HR-related gene; Region = region of the gene covered by the panel, E = exon.

## Supplementary Table S3

Table S3a: Somatic mutations detected in 103 patients of the EUDARIO study at the initiation of treatment.

Table S3b: Germline HRD mutations detected in whole blood from 103 patients of the EUDARIO study at the initiation of treatment.

Please see file SupplementaryTableS3.xlsx

## Supplementary Table S4a

Table S4a: Multivariate logistic regression with presence of at least one *DNMT3A*, *TET2* or *ASXL1* mutation (DTA) as dependent variable, and age decade, number of previous therapy lines (1 *vs* > 1) and duration of prior PARPi treatment in months as independent variables.

| Parameter | Level | Coefficient | Odds ratio | Lower 95%-CI | Upper 95%-CI | p-value |
| --- | --- | --- | --- | --- | --- | --- |
| Age decade |  | 0.67 | 1.95 | 1.21 | 3.12 | 0.006 |
| No. of previous therapy lines | >1 *vs* 1 | 0.40 | 1.50 | 0.53 | 4.20 | 0.442 |
| Duration of prior PARPi treatment in months |  | 0.05 | 1.05 | 0.99 | 1.12 | 0.125 |

## Supplementary Table S4b

Table S4b: Multivariate logistic regression with presence of at least one DDR mutation (*PPM1D, TP53, ATM* or *CHEK2*) as dependent variable, and age decade, number of previous therapy lines (1 *vs* > 1) and duration of prior PARPi treatment in months as independent variables.

| Parameter | Level | Coefficient | Odds ratio | Lower 95%-CI | Upper 95%-CI | p-value |
| --- | --- | --- | --- | --- | --- | --- |
| Age decade |  | 0.68 | 1.98 | 1.19 | 3.27 | 0.008 |
| No. of previous therapy lines | >1 *vs* 1 | 1.15 | 3.18 | 1.12 | 9.10 | 0.030 |
| Duration of prior PARPi treatment in months |  | 0.08 | 1.09 | 1.01 | 1.17 | 0.032 |

## Supplementary Table S5a

Table S5a: Occurrence of adverse events according to CH status

| Adverse event | level | CH negative  n = 50 | CH positive  n = 53 | p-value* |
| --- | --- | --- | --- | --- |
| Hematotoxicity – No. (%) | No | 6 (12.0) | 10 (18.9) | 0.419 |
|  | Yes | 44 (88.0) | 43 (81.1) |  |
| Neutropenia – No. (%) | No | 13 (26.0) | 23 (43.4) | 0.098 |
|  | Yes | 37 (74.0) | 30 (56.6) |  |
| Thrombocytopenia – No. (%) | No | 14 (28.0) | 24 (45.3) | 0.102 |
|  | Yes | 36 (72.0) | 29 (54.7) |  |
| Anemia – No. (%) | No | 14 (28.0) | 23 (43.4) | 0.150 |
|  | Yes | 36 (72.0) | 30 (56.6) |  |
| Infection – No. (%) | No | 37 (74.0) | 27 (50.9) | 0.025 |
|  | Yes | 13 (26.0) | 26 (49.1) |  |
| Allergic Reaction – No. (%) | No | 40 (80.0) | 36 (67.9) | 0.185 |
|  | Yes | 10 (20.0) | 17 (32.1) |  |
| Elevated transaminases – No. (%) | No | 39 (78.0) | 43 (81.1) | 0.808 |
|  | Yes | 11 (22.0) | 10 (18.9) |  |
| Acute kidney failure – No. (%) | No | 43 (86.0) | 49 (92.5) | 0.350 |
|  | Yes | 7 (14.0) | 4 (7.5) |  |
| Bleeding – No. (%) | No | 44 (88.0) | 50 (94.3) | 0.310 |
|  | Yes | 6 (12.0) | 3 (5.7) |  |

*p-value from Fisher’s exact test. Hematotoxicity is defined as the occurrence of anemia, neutropenia or thrombocytopenia.

## Supplementary Table S5b

Table S5b: Occurrence of adverse events during Carboplatin treatment according to CH status

| Adverse event | level | CH negative  n = 50 | CH positive  n = 53 | p-value* |
| --- | --- | --- | --- | --- |
| Hematotoxicity – No. (%) | No | 6 (12.0) | 11 (20.8) | 0.292 |
|  | Yes | 44 (88.0) | 42 (79.2) |  |
| Neutropenia – No. (%) | No | 14 (28.0) | 23 (43.4) | 0.150 |
|  | Yes | 36 (72.0) | 30 (56.6) |  |
| Thrombocytopenia – No. (%) | No | 15 (30.0) | 26 (49.1) | 0.070 |
|  | Yes | 35 (70.0) | 27 (50.9) |  |
| Anemia – No. (%) | No | 15 (30.0) | 24 (45.3) | 0.155 |
|  | Yes | 35 (70.0) | 29 (54.7) |  |
| Severe Hematotoxicity – No. (%) | No | 23 (46.0) | 29 (54.7) | 0.433 |
|  | Yes | 27 (54.0) | 24 (45.3) |  |
| Infection – No. (%) | No | 41 (82.0) | 36 (67.9) | 0.117 |
|  | Yes | 9 (18.0) | 17 (32.1) |  |
| Sever Infection – No. (%) | No | 48 (96.0) | 53 (100.0) | 0.233 |
|  | Yes | 2 (4.0) | 0 (0.0) |  |
| Allergic Reaction – No. (%) | No | 40 (80.0) | 36 (67.9) | 0.185 |
|  | Yes | 10 (20.0) | 17 (32.1) |  |
| Severe Allergic Reaction – No. (%) | No | 46 (92.0) | 52 (98.1) | 0.196 |
|  | Yes | 4 (8.0) | 1 (1.9) |  |
| Bleeding – No. (%) | No | 45 (90.0) | 50 (94.3) | 0.480 |
|  | Yes | 5 (10.0) | 3 (5.7) |  |
| Kidney Failure – No. (%) | No | 49 (98.0) | 52 (98.1) | 1.000 |
|  | Yes | 1 (2.0) | 1 (1.9) |  |
| Transaminases elevated – No. (%) | No | 40 (80.0) | 45 (84.9) | 0.607 |
|  | Yes | 10 (20.0) | 8 (15.1) |  |

*p-value from Fisher’s exact test. Hematotoxicity is defined as the occurrence of anemia, neutropenia or thrombocytopenia.

## Supplementary Table S5c

Table S5c: Occurrence of adverse events during niraparib maintenance treatment according to CH status

| Adverse event | level | CH negative  n = 34 | CH positive  n = 34 | p-value* |
| --- | --- | --- | --- | --- |
| Hematotoxicity – No. (%) | No | 12 (35.3) | 15 (44.1) | 0.621 |
|  | Yes | 22 (64.7) | 19 (55.9) |  |
| Neutropenia – No. (%) | No | 20 (58.8) | 23 (67.6) | 0.615 |
|  | Yes | 14 (41.2) | 11 (32.4) |  |
| Thrombocytopenia – No. (%) | No | 21 (61.8) | 23 (67.6) | 0.800 |
|  | Yes | 13 (38.2) | 11 (32.4) |  |
| Anemia – No. (%) | No | 18 (52.9) | 22 (64.7) | 0.460 |
|  | Yes | 16 (47.1) | 12 (35.3) |  |
| Severe Hematotoxicity – No. (%) | No | 23 (67.6) | 24 (70.6) | 1.000 |
|  | Yes | 11 (32.4) | 10 (29.4) |  |
| Infection – No. (%) | No | 27 (79.4) | 22 (64.7) | 0.280 |
|  | Yes | 7 (20.6) | 12 (35.3) |  |
| Sever Infection – No. (%) | No | 33 (97.1) | 33 (97.1) | 1.000 |
|  | Yes | 1 (2.9) | 1 (2.9) |  |
| Allergic Reaction – No. (%) | No | 34 (100.0) | 34 (100.0) | NA |
|  | Yes | 0 (0.0) | 0 (0.0) |  |
| Severe Allergic Reaction – No. (%) | No | 34 (100.0) | 34 (100.0) | NA |
|  | Yes | 0 (0.0) | 0 (0.0) |  |
| Bleeding – No. (%) | No | 33 (97.1) | 33 (97.1) | 1.000 |
|  | Yes | 1 (2.9) | 1 (2.9) |  |
| Kidney Failure – No. (%) | No | 28 (82.4) | 31 (91.2) | 0.476 |
|  | Yes | 6 (17.6) | 3 (8.8) |  |
| Transaminases elevated – No. (%) | No | 31 (91.2) | 32 (94.1) | 1.000 |
|  | Yes | 3 (8.8) | 2 (5.9) |  |

*p-value from Fisher’s exact test. Hematotoxicity is defined as the occurrence of anemia, neutropenia or thrombocytopenia.

## Supplementary Table S6

Table S6: Interruption, dose reduction or discontinuation of study treatment due to any cytopenia according to CH status for Niraparib maintenance, carboplatin or during any phase of the treatment. ‘Any event’ includes interruption, dose reduction, or discontinuation.

|  |  | level | CH negative  n = 50 | CH positive  n = 53 | p-value* |
| --- | --- | --- | --- | --- | --- |
| Niraparib  maintenance | Interruption – No. (%) | No | 38 (76.0) | 43 (81.1) | 0.632 |
|  |  | Yes | 12 (24.0) | 10 (18.9) |  |
|  | Dose reduction – No. (%) | No | 39 (78.0) | 43 (81.1) | 0.808 |
|  |  | Yes | 11 (22.0) | 10 (18.9) |  |
|  | Any event – No. (%) | No | 33 (66.0) | 38 (71.7) | 0.670 |
|  |  | Yes | 17 (34.0) | 15 (28.3) |  |
| Carboplatin | Interruption – No. (%) | No | 18 (36.0) | 24 (45.3) | 0.423 |
|  |  | Yes | 32 (64.0) | 29 (54.7) |  |
|  | Dose reduction – No. (%) | No | 41 (82.0) | 43 (81.1) | 1.000 |
|  |  | Yes | 9 (18.0) | 10 (18.9) |  |
|  | Any event – No. (%) | No | 15 (30.0) | 23 (43.4) | 0.220 |
|  |  | Yes | 35 (70.0) | 30 (56.6) |  |
| Overall treatment | Interruption – No. (%) | No | 11 (22.0) | 21 (39.6) | 0.059 |
|  |  | Yes | 39 (78.0) | 32 (60.4) |  |
|  | Dose reduction – No. (%) | No | 29 (58.0) | 32 (60.4) | 0.843 |
|  |  | Yes | 21 (42.0) | 21 (39.6) |  |
|  | Discontinuation – No. (%) | No | 43 (86.0) | 51 (96.2) | 0.087 |
|  |  | Yes | 7 (14.0) | 2 (3.8) |  |

*p-value from Fisher’s exact test.

## Supplementary Table S7

Table S6: Best response to treatment according to CH status

| Best response – No. (%) | CH negative  n = 50 | CH positive  n = 53 |
| --- | --- | --- |
| CR | 12 (24.0) | 7 (13.2) |
| PR | 11 (22.0) | 14 (26.4) |
| SD | 17 (34.0) | 19 (35.8) |
| PD | 8 (16.0) | 10 (18.9) |
| NA | 2 (4.0) | 3 (5.7) |

CR = complete remission, PD = progressive disease, PR = partial remission, SD = stable disease, NA = not applicable (patients died before first response evaluation)

## Supplementary Table S8a

Table S8a: Occurrence of hematoxicity stratified by HRD germline status during carboplatin treatment.

| Adverse event | level | HRD negative  n = 72 | HRD positive  n = 31 | p-value* |
| --- | --- | --- | --- | --- |
| Hematotoxicity – No. (%) | No | 14 (19.4) | 3 (9.7) | 0.262 |
|  | Yes | 58 (80.6) | 28 (90.3) |  |
| Neutropenia – No. (%) | No | 27 (37.5) | 10 (32.3) | 0.660 |
|  | Yes | 45 (62.5) | 21 (67.7) |  |
| Thrombocytopenia – No. (%) | No | 35 (48.6) | 6 (19.4) | **0.008** |
|  | Yes | 37 (51.4) | 25 (80.6) |  |
| Anemia – No. (%) | No | 27 (37.5) | 12 (38.7) | 1.000 |
|  | Yes | 45 (62.5) | 19 (61.3) |  |
| Any severe hematotoxicity – No. (%) | No | 35 (48.6) | 17 (54.8) | 0.669 |
|  | Yes | 37 (51.4) | 14 (45.2) |  |

*p-value from Fisher’s exact test; hematotoxicity is defined as the occurrence of any cytopenia. Severe hematotoxicity is defined as neutropenia, thrombocytopenia or anemia of grade ≥ 3 according to the common terminology criteria for adverse events (CTCAE)

## Supplementary Table S8b

Table S8b: Occurrence of hematoxicity stratified by HRD germline status during niraparib maintenance.

| Adverse event | level | HRD negative  n = 46 | HRD positive  n = 22 | p-value* |
| --- | --- | --- | --- | --- |
| Hematotoxicity – No. (%) | No | 18 (39.1) | 9 (40.9) | 1.000 |
|  | Yes | 28 (60.9) | 13 (59.1) |  |
| Neutropenia – No. (%) | No | 30 (65.2) | 13 (59.1) | 0.825 |
|  | Yes | 16 (34.8) | 9 (40.9) |  |
| Thrombocytopenia – No. (%) | No | 29 (63.0) | 15 (68.2) | 0.886 |
|  | Yes | 17 (37.0) | 7 (31.8) |  |
| Anemia – No. (%) | No | 26 (56.5) | 14 (63.6) | 0.769 |
|  | Yes | 20 (43.5) | 8 (36.4) |  |
| Any severe hematotoxicity – No. (%) | No | 33 (71.7) | 14 (63.6) | 0.692 |
|  | Yes | 13 (28.3) | 8 (36.4) |  |

*p-value from Fisher’s exact test; hematotoxicity is defined as the occurrence of any cytopenia. Severe hematotoxicity is defined as neutropenia, thrombocytopenia or anemia of grade ≥ 3 according to the common terminology criteria for adverse events (CTCAE)

## Supplementary Table S9a

Table S9a: Interruption, dose reduction or discontinuation of study treatment according to HRD germline status for niraparib maintenance, carboplatin treatment, or during any phase of the treatment due to any cytopenia.

|  |  | level | HRD negative  n = 72 | HRD positive  n = 31 | p-value* |
| --- | --- | --- | --- | --- | --- |
| Overall treatment | Interruption – No. (%) | No | 26 (36.1) | 6 (19.4) | 0.108 |
|  |  | Yes | 46 (63.9) | 25 (80.6) |  |
|  | Dose reduction – No. (%) | No | 45 (62.5) | 16 (51.6) | 0.383 |
|  |  | Yes | 27 (37.5) | 15 (48.4) |  |
|  | Discontinuation – No. (%) | No | 66 (91.7) | 28 (90.3) | 1.000 |
|  |  | Yes | 6 (8.3) | 3 (9.7) |  |

*p-value from Fisher’s exact test.

## Supplementary Table S9b

Table S9b: Interruption, dose reduction or discontinuation of study treatment according to HRD germline status for niraparib maintenance, carboplatin treatment, or during any phase of the treatment due to thrombocytopenia.

|  |  | level | HRD negative  n = 72 | HRD positive  n = 31 | p-value* |
| --- | --- | --- | --- | --- | --- |
| Overall treatment | Interruption – No. (%) | No | 45 (62.5) | 15 (48.4) | 0.198 |
|  |  | Yes | 27 (37.5) | 16 (51.6) |  |
|  | Discontinuation – No. (%) | No | 69 (95.8) | 30 (96.8) | 1.000 |
|  |  | Yes | 3 (4.2) | 1 (3.2) |  |
|  | Dose reduction – No. (%) | No | 56 (77.8) | 25 (80.6) | 0.800 |
|  |  | Yes | 16 (22.2) | 6 (19.4) |  |

*p-value from Fisher’s exact test.

## Supplementary Table S10

Table S7: Clinical characteristics of 37 patients with OC undergoing PARPi treatment, prospectively recruited to obtain material for single-cell genotyping.

| Characteristic | Level | CH negative  n = 7 | CH positive  n = 30 | p-value* |
| --- | --- | --- | --- | --- |
| Age – median (IQR) |  | 59 (51 - 65) | 65 (57 - 73) | 0.09 |
| Germline *BRCA* status – No. (%) | Mutated | 3 (57) | 8 (27) | 0.40 |
|  | Wildtype | 4 (43) | 22 (73) |  |
| History of cancer – No. (%) | No | 7 (100) | 27 (93) | 1 |
|  | Yes | 0 (0) | 2 (7) |  |
| Number of previous lines – No. (%) | 1 | 6 (86) | 11 (37) | 0.033 |
|  | >1 | 1 (14) | 19 (63) |  |
| Duration of prior PARPi treatment in months – median (IQR) |  | 9 (5 - 11) | 11 (6 - 24) | 0.34 |

IQR – interquartile range, *p-value from Fisher’s exact test or Wilcoxon rank sum test.

## Supplementary Table S11

Table S8: Overview of MissionBio readout from 11 samples subjected to single-cell DNA sequencing on the MissionBio Tapestri platform. Samples of Type “mixed” were pooled and demultiplexed using a predetermined set of individual SNPs as described in the Supplementary Methods section. The panel was chosen based on the optimal coverage of somatic mutations determined by bulk sequencing.


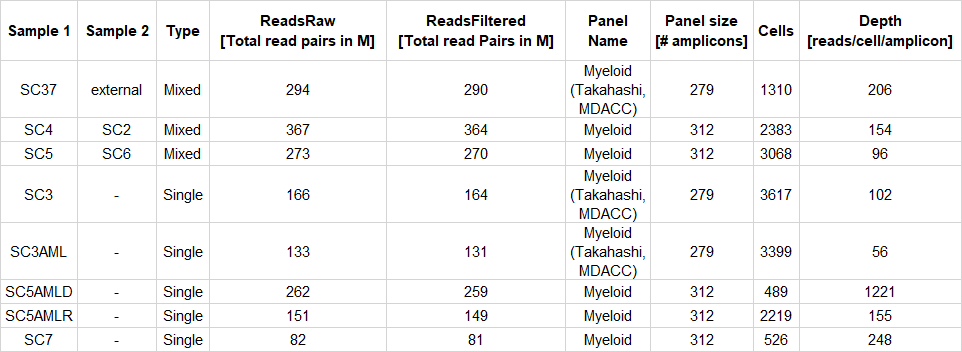


# Supplementary Figures

## Supplementary Figure S1a


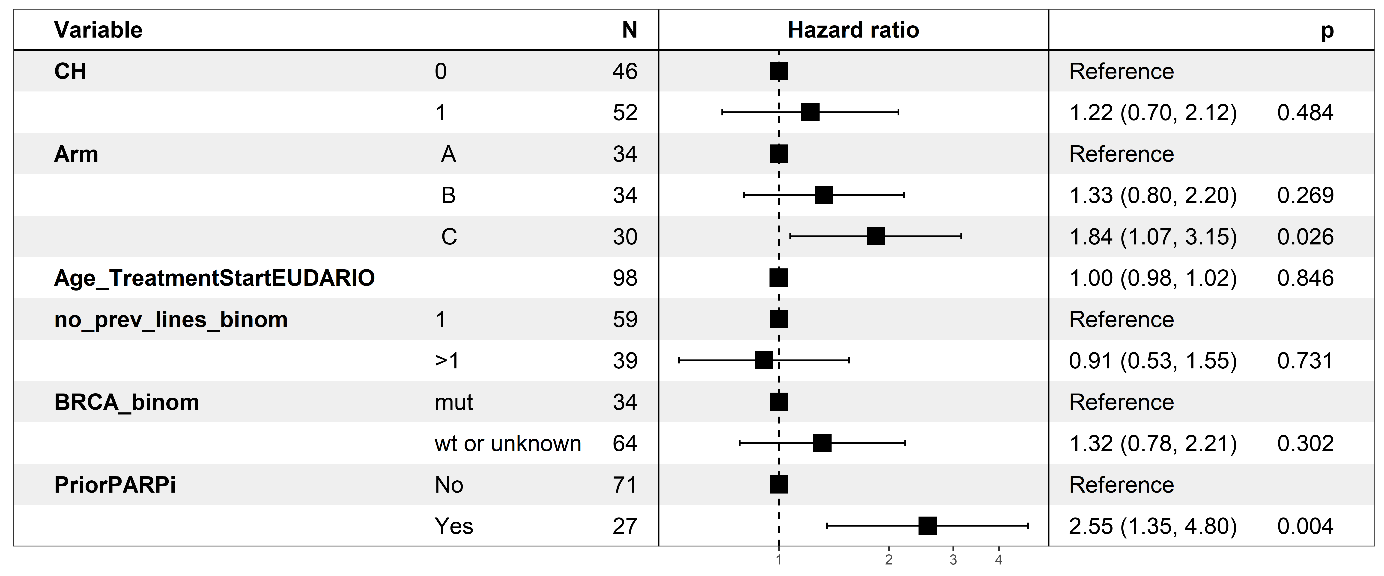


Figure S1a: Multivariate Cox regression model with progression-free survival (PFS) as dependent, and study arm (Arm), age (Age_TreatmentStartEUDARIO), number of previous treatment lines (no_prev_lines_binom), *BRCA* status (BRCA_status) and prior PARPi exposure (PriorPARPi) as independent variables.

## Supplementary Figure S1b


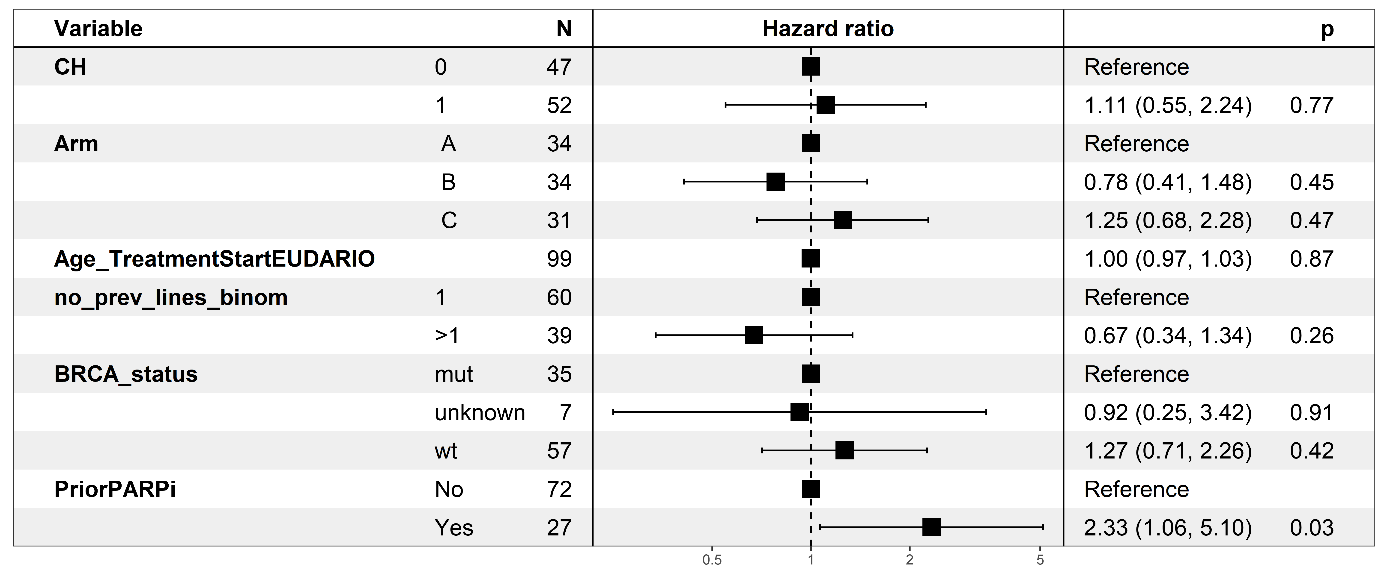


Figure S1b: Multivariate Cox regression model with overall survival (OS) as dependent, and study arm (Arm), age (Age_TreatmentStartEUDARIO), number of previous treatment lines (no_prev_lines_binom), *BRCA* status (BRCA_status) and prior PARPi exposure (PriorPARPi) as independent variables.

## Supplementary Figure S2


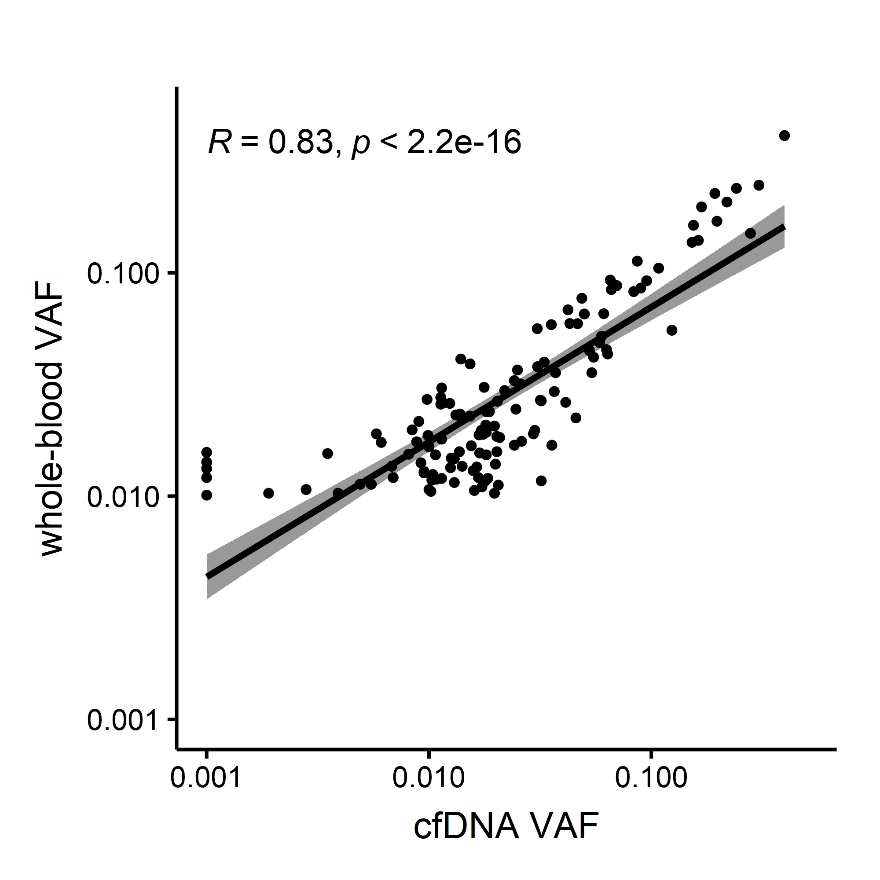


Figure S2: Scatterplot of VAFs in WB DNA vs VAFs in cfDNA of 130 somatic mutations detected in WB DNA with VAF ≥ 1% of 103 patients participating in the EUDARIO study. R is the Pearson correlation coefficient.

## Supplementary Figure S3


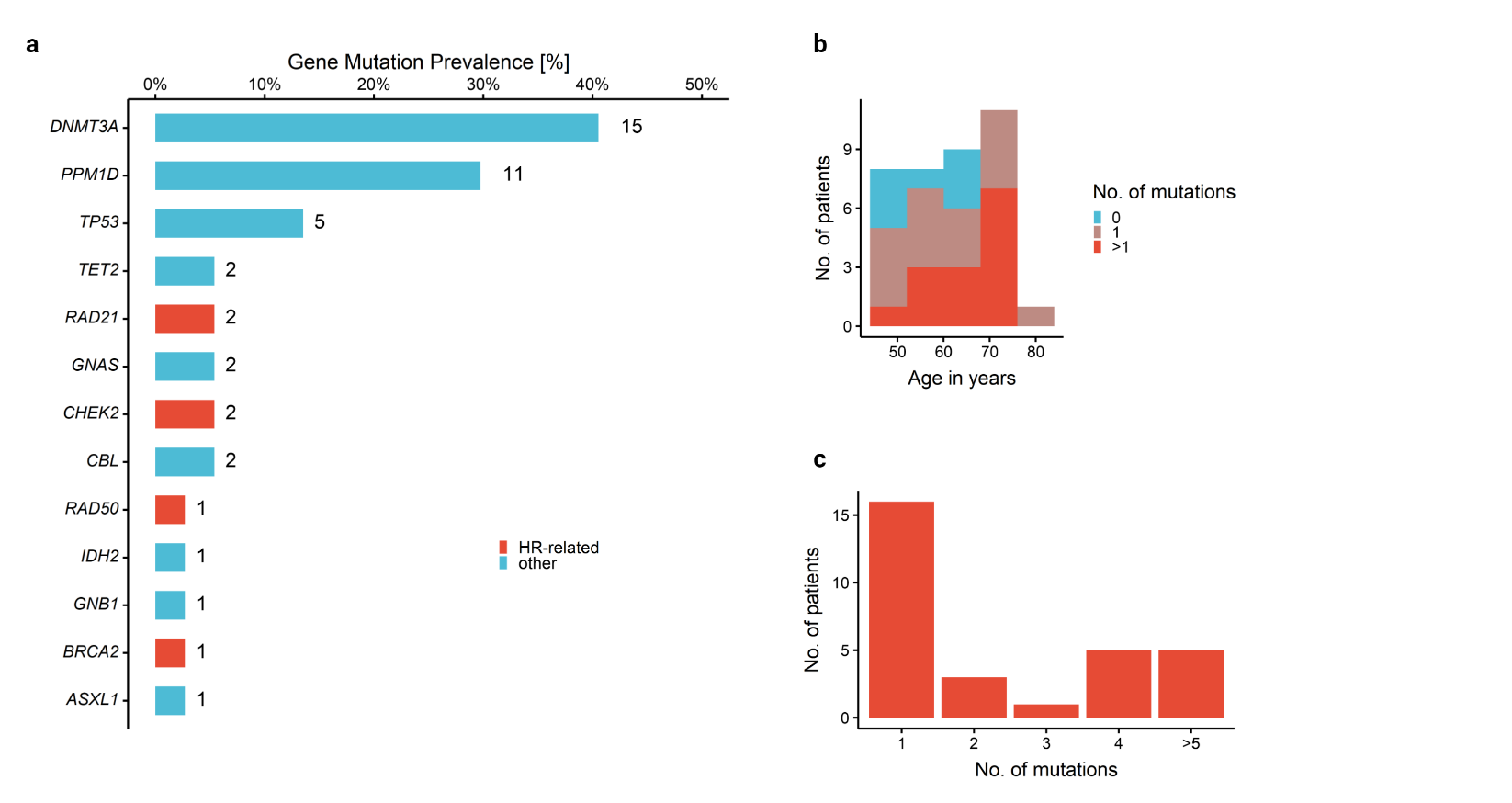


Figure S3: Somatic mutations in WB DNA from 37 patients with HGOC undergoing PARPi and/or Platinum-based treatment. a) Gene-specific prevalence of CH. b) Age distribution of the 37 patients according to CH status (no CH, CH with single mutation, CH with multiple mutations). c) Prevalence of CH according to number of mutations.

# References

1. Goldman JW, Raju RN, Gordon GA, El-Hariry I, Teofilivici F, Vukovic VM, et al. A first in human, safety, pharmacokinetics, and clinical activity phase I study of once weekly administration of the Hsp90 inhibitor ganetespib (STA-9090) in patients with solid malignancies. *BMC Cancer*. 2013;13:152.

2. Arends CM, Galan-Sousa J, Hoyer K, Chan W, Jager M, Yoshida K, et al. Hematopoietic lineage distribution and evolutionary dynamics of clonal hematopoiesis. *Leukemia*. 2018;32(9):1908-1919.

3. Molder F, Jablonski KP, Letcher B, Hall MB, Tomkins-Tinch CH, Sochat V, et al. Sustainable data analysis with Snakemake. *F1000Res*. 2021;10:33.

4. Arends CM, Dimitriou S, Stahler A, Hablesreiter R, Strzelecka PM, Stein CM, et al. Clonal hematopoiesis is associated with improved survival in patients with metastatic colorectal cancer from the FIRE-3 trial. *Blood*. 2022;139(10):1593-1597.

5. Arends CM, Liman TG, Strzelecka PM, Kufner A, Lowe P, Huo S, et al. Associations of clonal hematopoiesis with recurrent vascular events and death in patients with incident ischemic stroke. *Blood*. 2023;141(7):787-799.

6. Li H, Durbin R. Fast and accurate long-read alignment with Burrows-Wheeler transform. *Bioinformatics*. 2010;26(5):589-595.

7. DePristo MA, Banks E, Poplin R, Garimella KV, Maguire JR, Hartl C, et al. A framework for variation discovery and genotyping using next-generation DNA sequencing data. *Nat Genet*. 2011;43(5):491-498.

8. McKenna A, Hanna M, Banks E, Sivachenko A, Cibulskis K, Kernytsky A, et al. The Genome Analysis Toolkit: a MapReduce framework for analyzing next-generation DNA sequencing data. *Genome Res*. 2010;20(9):1297-1303.

9. Lai Z, Markovets A, Ahdesmaki M, Chapman B, Hofmann O, McEwen R, et al. VarDict: a novel and versatile variant caller for next-generation sequencing in cancer research. *Nucleic Acids Res*. 2016;44(11):e108.

10. Wang K, Li M, Hakonarson H. ANNOVAR: functional annotation of genetic variants from high-throughput sequencing data. *Nucleic Acids Res*. 2010;38(16):e164.

11. O'Leary NA, Wright MW, Brister JR, Ciufo S, Haddad D, McVeigh R, et al. Reference sequence (RefSeq) database at NCBI: current status, taxonomic expansion, and functional annotation. *Nucleic Acids Res*. 2016;44(D1):D733-745.

12. Karczewski KJ, Francioli LC, Tiao G, Cummings BB, Alfoldi J, Wang Q, et al. The mutational constraint spectrum quantified from variation in 141,456 humans. *Nature*. 2020;581(7809):434-443.

13. Sherry ST, Ward M, Sirotkin K. dbSNP-database for single nucleotide polymorphisms and other classes of minor genetic variation. *Genome Res*. 1999;9(8):677-679.

14. Tate JG, Bamford S, Jubb HC, Sondka Z, Beare DM, Bindal N, et al. COSMIC: the Catalogue Of Somatic Mutations In Cancer. *Nucleic Acids Res*. 2019;47(D1):D941-D947.

15. Robinson JT, Thorvaldsdottir H, Winckler W, Guttman M, Lander ES, Getz G, et al. Integrative genomics viewer. *Nat Biotechnol*. 2011;29(1):24-26.

16. Gerstung M, Pellagatti A, Malcovati L, Giagounidis A, Porta MG, Jadersten M, et al. Combining gene mutation with gene expression data improves outcome prediction in myelodysplastic syndromes. *Nat Commun*. 2015;6:5901.

17. Morita K, Wang F, Jahn K, Hu T, Tanaka T, Sasaki Y, et al. Clonal evolution of acute myeloid leukemia revealed by high-throughput single-cell genomics. *Nat Commun*. 2020;11(1):5327.

18. Sollier E, Kuipers J, Takahashi K, Beerenwinkel N, Jahn K. COMPASS: joint copy number and mutation phylogeny reconstruction from amplicon single-cell sequencing data. *Nat Commun*. 2023;14(1):4921.

19. Borgsmuller N, Bonet J, Marass F, Gonzalez-Perez A, Lopez-Bigas N, Beerenwinkel N. BnpC: Bayesian non-parametric clustering of single-cell mutation profiles. *Bioinformatics*. 2020;36(19):4854-4859.
